# A model of the oscillatory mechanical forces in the conventional outflow pathway

**DOI:** 10.1098/rsif.2018.0652

**Published:** 2019-01-30

**Authors:** Joseph M. Sherwood, W. Daniel Stamer, Darryl R. Overby

**Affiliations:** 1 Department of Bioengineering, Imperial College London, London, UK; 2 Department of Ophthalmology, Duke University, Durham, NC, USA

**Keywords:** outflow resistance, intraocular pressure, Schlemm's canal, trabecular meshwork, glaucoma

## Abstract

Intraocular pressure is regulated by mechanosensitive cells within the conventional outflow pathway, the primary route of aqueous humour drainage from the eye. However, the characteristics of the forces acting on those cells are poorly understood. We develop a model that describes flow through the conventional outflow pathway, including the trabecular meshwork (TM) and Schlemm’s canal (SC). Accounting for the ocular pulse, we estimate the time-varying shear stress on SC endothelium and strain on the TM. We consider a range of outflow resistances spanning normotensive to hypertensive conditions. Over this range, the SC shear stress increases significantly and becomes highly oscillatory. TM strain also increases, but with negligible oscillations. Interestingly, TM strain responds more to changes in outflow resistance around physiological values, while SC shear stress responds more to elevated levels of resistance. A modest increase in TM stiffness, as observed in glaucoma, suppresses TM strain and practically eliminates the influence of outflow resistance on SC shear stress. As SC and TM cells respond to mechanical stimulation by secreting factors that modulate outflow resistance, our model provides insight regarding the potential role of SC shear and TM strain as mechanosensory cues for homeostatic regulation of outflow resistance and hence intraocular pressure.

## 1. Introduction

The conventional outflow pathway is the primary route of aqueous humour drainage from the eye. Hydraulic resistance generated within the outflow pathway determines intraocular pressure (IOP), which maintains the ocular shape necessary for visual acuity. In healthy individuals, IOP lies within relatively narrow bounds without significant differences between young and old populations [1,2], implying some degree of homeostatic regulation. In glaucoma, IOP often becomes elevated, correlating with increased outflow resistance [3]. Elevated IOP damages optic nerve axons to cause glaucomatous blindness, and all treatments for glaucoma aim to lower IOP. It is thus important to understand the mechanisms controlling IOP and how these mechanisms become disrupted in glaucoma.

**Figure 1.**
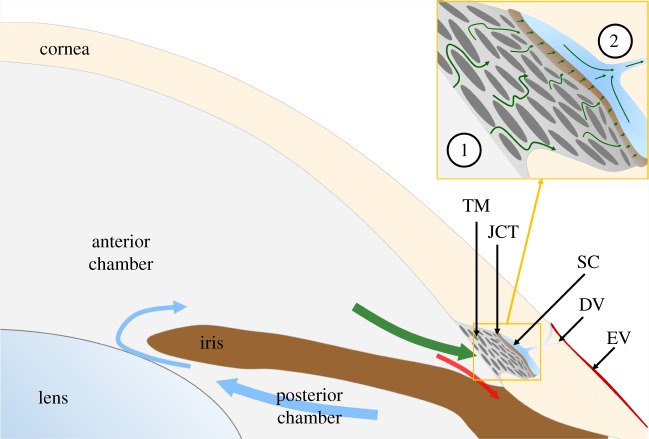
Aqueous humour is secreted into the posterior chamber at a nearly constant rate, enters the anterior chamber through the pupil (blue arrows) and then drains through one of two outflow pathways. The conventional outflow pathway (green arrow) includes the trabecular meshwork (TM), juxtacanalicular tissue (JCT), Schlemm’s canal (SC) and distal vessels (DV), comprising collector channels and intrascleral vessels, leading to episcleral vessels (EV). The unconventional pathway includes flow through the iris root (red arrow) and is believed to be relatively pressure-independent. The circled numbers in the inset refer to the location of the anterior chamber and collector channels shown in figure 2.

Aqueous humour passing through the conventional outflow pathway first encounters the trabecular meshwork (TM) that contains collagenous beams lined by TM cells (figure 1). Between the TM and Schlemm's canal (SC). lies the juxtacanalicular tissue (JCT), a thin zone of loose connective tissue containing TM-like cells. Downstream of the TM/JCT is SC, an endothelial-lined vessel that collects aqueous humour. Aqueous humour in SC flows through collector channels (CC) and intrascleral vessels (collectively referred to as distal vessels, DV) that provide a pathway across the sclera to the episcleral vessels, where aqueous humour returns to the systemic circulation. The bulk of outflow resistance is believed to reside within the JCT and inner wall endothelium of SC [4,5], with additional resistance generated within the CC and intrascleral vessels [6].

In this study, we build on a previous mathematical model [7] to estimate the mechanical stretch or ‘strain’ on the TM tissue as a whole and the shear stress arising from circumferential flow through SC in human eyes. These mechanical cues may be involved in IOP homeostasis because TM and SC cells respond to stretch and shear stress by secreting factors that modulate outflow resistance, including matrix metalloproteases (MMPs) [8], adenosine [9], nitric oxide (NO) [10] and vascular endothelial growth factor (VEGF) [11]. We account for the elasticity of the TM that expands with increasing IOP and narrows SC, thereby affecting SC shear stress. We also introduce to the model the role of oscillations in IOP, known as the *ocular pulse*, induced by cardiac pulsations in the choroid and retina, as well as pressure oscillations in the episcleral vessels. We examine how the magnitude of oscillatory TM strain and SC shear stress depends on outflow resistance and IOP, thereby allowing for mechanosensation that is a necessary condition for IOP homeostasis. Finally, we investigate how these mechanical cues may be disrupted by increased TM stiffness that may occur in glaucoma [12,13].

## 2. Model formulation

The model used in this study builds on the one-dimensional TM/SC model of Johnson & Kamm [7]. Here, we make four major modifications: (i) we couple the TM/SC model to a lumped parameter model representing the rest of the eye; (ii) we consider an ‘*in vivo*’ paradigm, in which the net flow through the conventional outflow pathway is constant; (iii) we account for the nonlinear pressure–height relationship of SC; and (iv) we consider the oscillatory nature of the flow and resulting mechanical forces in the conventional outflow pathway due to internal and external pressure oscillations. For brevity, we provide only a brief description of the model formulation here. Full details can be found in the electronic supplementary material.

### 2.1. Lumped parameter model of the whole eye

**Figure 2.**
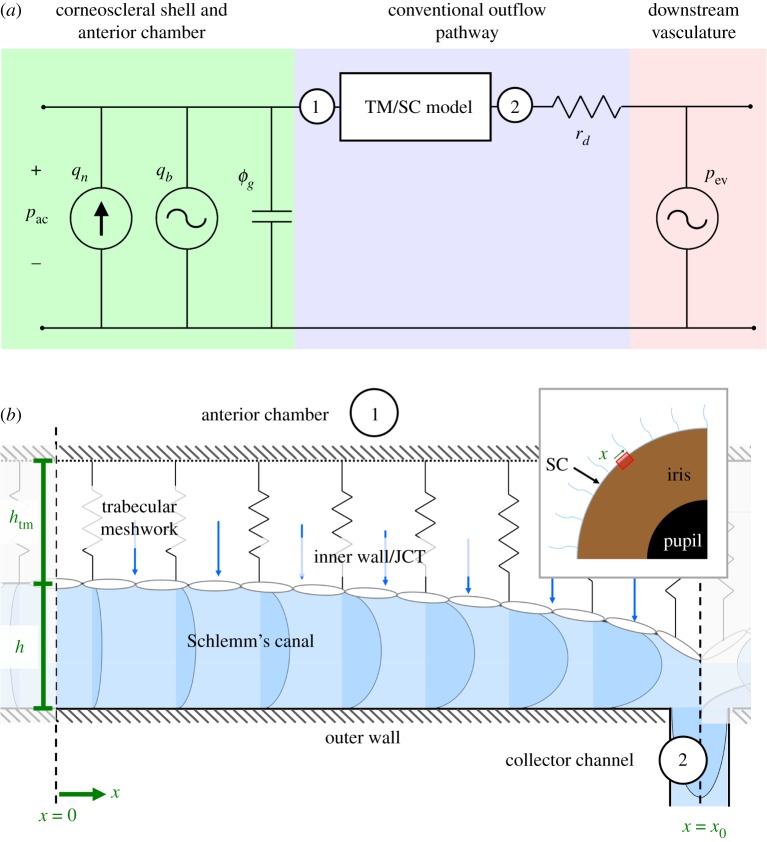
(*a*) Coupling the TM/SC model to a lumped parameter model of the whole eye. Circled numbers represent the locations where the TM/SC model interfaces at the anterior chamber ① and collector channels ②. (*b*) A schematic of an individual segment of the TM/SC model. Inset: frontal view of the eye indicating the mathematical domain (red rectangle) relative to collector channels (blue), iris (brown) and pupil (black).

In order to model the interaction between flow through the outflow pathway and fluid volume in the rest of the eye, we couple the TM/SC model (§2.2) to the lumped parameter model shown in figure 2*a*. Upstream of the TM/SC, there is: a steady flow source *q*_*n*_, representing the net pressure-independent aqueous humour flow (the difference between the rate of aqueous humour secretion and any pressure-independent outflow); an oscillatory flow source *q*_*b*_, representing the flow of blood into and out of the intraocular vasculature during the cardiac cycle (which has zero mean); and the pressure-dependent compliance of the corneoscleral globe *ϕ*_*g*_. Downstream of the TM/SC, *r*_*d*_ represents the hydrodynamic resistance of the DV that allow flow from SC to the episcleral vessels. Episcleral vessel pressure (EVP), *p*_ev_, is modelled as an oscillatory pressure source, following studies showing the oscillatory nature of EVP *in vivo* [14,15].

### 2.2. Model of the TM and SC

A schematic of a segment of the TM and SC, based on the model of Johnson & Kamm [7], is shown in figure 2*b*. The circumferential segment is defined between *x* = 0 at a point equidistant between CCs and *x* = *x*_0_ at the CC ostium (note that *x* refers to the out-of-plane coordinate in figure 1). The height of SC is *h*, and the height of the TM is *h*_tm_, where *h* + *h*_tm_ is constant, consistent with observations in human imaging studies [16]. The values of *h* and *h*_tm_ are determined by the apparent stiffness of the TM, and the pressure drop across the inner wall/JCT, defined as the pressure difference between anterior chamber (*p*_ac_), and SC (*p*_sc_), which is a function of *x* and time. Aqueous humour crosses the inner wall/JCT and flows circumferentially along SC lumen to reach the CC ostium. There are 2*n* such segments in parallel making up the complete circumference of the TM and SC around the entire eye, where *n* is the number of CC ostia. The inset shows a frontal view of the eye, with the region covered by figure 1*b* indicated in red.

#### 2.2.1. Summary of the fundamental equations

The model of the TM and SC is formulated using four equations, which describe:

(1) The filtration of aqueous humour across the TM and inner wall, determined by the pressure difference between the anterior chamber and SC, *p*_ac_ − *p*_sc_, and the resistance generated by the inner wall/JCT, *r*_*je*_.
(2) The expansion of the TM, modelled as a spring, such that an increase in the pressure difference across the TM results in a reduction in SC height. Rather than defining a single value of TM elastic modulus, the range of which spans several orders of magnitude in the literature [17], we define an apparent TM stiffness based on observations of SC height at different values of IOP. This approach captures the nonlinear pressure–height relationship of SC.
(3) The conservation of aqueous humour mass within SC. The flow rate *q* in SC varies as a function of *x* and time, due to filtration across the inner wall and time-dependent changes in SC height.
(4) The pressure gradient along SC due to viscous dissipation. This pressure gradient is proportional to the product of *q* and the resistance to flow in SC, *r*_sc_, approximating SC as a wide rectangular channel. *r*_sc_ depends on *h*, *w*, *x*_0_ and the viscosity of aqueous humour, which is assumed to equal that of water.

These equations are combined to yield a single second-order, nonlinear partial differential equation describing SC height as a function of time and location along SC. This equation is solved using MATLAB subject to the following boundary conditions.

#### 2.2.2. Summary of the boundary and initial conditions

At *x* = 0, there is a plane of symmetry, such that a spatial gradient of SC height at this location is zero. At the CC ostium (*x* = *x*_0_), the pressure in SC is the sum of *p*_ev_ and the pressure drop across the CCs and intrascleral vessels. This condition is expressed in terms of SC height using the fundamental equations. The initial condition uses the steady state solution for the SC height for the case when *r*_sc_ = 0.

### 2.3. Independent parameter

This study uses the *in vivo* paradigm, in which the net flow rate through the system is constant. In this model, IOP elevation occurs due to an increase in resistance to outflow, the majority of which lies in the vicinity of SC inner wall. The independent parameter in our model is therefore the JCT/inner wall resistance *r*_*je*_.

Cell debris and pigment are transported to the TM by aqueous humour outflow, and phagocytosis by TM cells is responsible for clearing away this debris. Furthermore, TM cells are responsible for matrix remodelling via production of both lytic enzymes and extracellular matrix (ECM). The extent of debris and matrix accumulation in the TM thereby influences outflow resistance, which we model by varying *r*_*je*_.

For all simulations, *r*_*je*_ is varied in the range of 0.08 to 7.71 mmHg/(μl min^−1^), corresponding to values of *p*_ac_ between 10.6 and 28.0 mmHg for the ‘normal’ parameters. A value of *r*_*je*_ = 2.08 mmHg/(μl min^−1^) corresponds to the normal inner wall/JCT resistance (i.e. consistent with the values given in the electronic supplementary material, table A2 with equation (3) under the assumption that $\tilde{r_{\mathrm{sc}}}$ is negligible).

### 2.4. Model coupling

Defining *r*_*je*_ as the independent parameter adds complexity because *p*_ac_ becomes an output from the model rather than an input (as considered by Johnson & Kamm [7]). Solving the fundamental equations requires knowledge of *p*_ac_ and yields a solution for the flow of aqueous humour through SC. However, due to the constancy of aqueous humour secretion, the total flow through SC is constrained. To address this issue, an iterative procedure is carried out for each value of *r*_*je*_, for which we must find the value of *p*_ac_ that yields the correct value of *q*_*n*_. The process involves three steps: (i) an initial guess of *p*_ac_ is used and a solution is found; (ii) the estimated value of *q*_*n*_ is compared against the target value, and a new estimate of *p*_ac_ is made based on this difference; (iii) the process is repeated until the target value of *q*_*n*_ is achieved, yielding the final converged solution. The desired data (shear stress in SC and the DV and the strain in the TM) can then be extracted for analysis.

### 2.5. Investigated parameter

The model is used to investigate the effect of *r*_*je*_ and the increase in TM stiffness thought to occur in glaucoma [12,13]. We examine the latter by multiplying the apparent TM stiffness, *ξ*, by a factor *n*_*ξ*_. We consider *n*_*ξ*_ = 1.0, 1.5 and 4 to roughly approximate the magnitude of the TM stiffness changes reported in the literature [12,13,18].

## 3. Results

### 3.1. Normal human parameters

**Figure 3.**
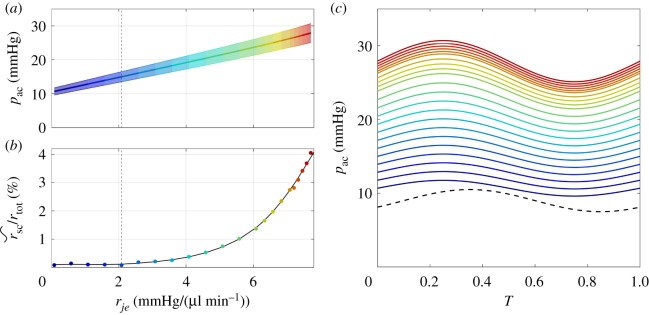
(*a*) Anterior chamber pressure *p*_ac_ versus inner wall/JCT resistance *r*_*je*_. The central line in (*a*) shows the time-averaged anterior chamber pressure, $\bar{\;p_{\mathrm{ac}}}$, while the shaded region shows the amplitude of the pressure oscillations. The colour scheme represents the value of *r*_*je*_ and is consistent with the colour scheme in (*c*) and in figure 4. (*b*) The relative fraction of total outflow resistance attributable to flow in Schlemm’s canal, $\tilde{r_{\mathrm{sc}}}/r_{\mathrm{tot}}\times 100\text{\%}$, increases as a function of *r*_*je*_. (*c*) Temporal variations in *p*_ac_(*T*) over one cardiac cycle increase with *r*_*je*_. Dashed black curve shows *p*_ev_(*T*).

The relationship between *p*_ac_ and *r*_*je*_ for normal human parameters is shown in figure 3*a*. As the inner wall/JCT resistance increases, the time-averaged AC pressure increases approximately linearly. This implies that flow resistance in SC does not contribute significantly to total outflow resistance. Indeed, the percentage of total resistance attributable to flow within SC ($\tilde{r_{\mathrm{sc}}}/r_{\mathrm{tot}}\times 100\text{\%}$) does not exceed approximately 4% (figure 3*b*). The amplitude of the pressure oscillations, *a*_ac_, indicated by the shading in figure 3*a*, increases linearly with *p*_ac_. Figure 3*c* shows the sinusoidal waveforms for *p*_ac_ and *p*_ev_, illustrating an imposed phase difference *ψ*_ev_. Note that the time axis is normalized by the cardiac cycle time to express in terms of non-dimensional time, *T*.

**Figure 4.**
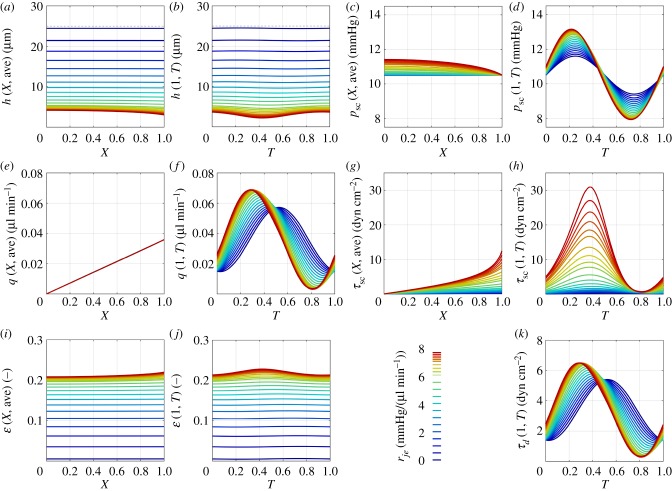
Model predictions shown as a function of *X* (time-averaged) and *T* (evaluated at *X* = 1) as inner wall/JCT resistance, *r*_*je*_, increases. (*a*,*b*) SC height, *h*, where dashed lines show the resting SC height *h*_0_. (*c*,*d*) The pressure in SC, *p*_sc_. (*e*,*f*) The flow rate in SC, *q*. (*g*,*h*) The shear stress acting on the endothelium of SC, *τ*_sc_. (*i*,*j*) The strain acting on the TM, *ɛ*. (*k*) The shear stress in the distal vessels, *τ*_*d*_. Coloured tracings indicate the value of *r*_*je*_, as shown in the legend between (*j*) and (*k*).

The temporal and spatial distributions of the key variables are summarized in figure 4. Figure 4*a* shows the time-averaged channel height along SC, which decreases for increasing *r*_*je*_, against *X* = *x*/*x*_0_ as the normalized channel position. For low *r*_*je*_, the channel height is relatively independent of *X*, but as *r*_*je*_ increases, SC height decreases near the CC ostium (*X* = 1). For the highest value of *r*_*je*_, the time-averaged SC height at the CC ostium is almost 30% lower than that at *X* = 0. The oscillation amplitude of *h* is also sensitive to *r*_*je*_ (figure 4*b*). For the highest value of *r*_*je*_, the peak-to-peak amplitude of *h* is 50% of its mean value, as compared to less than 1% for physiological *r*_*je*_.

The non-uniformity in *h* along *X* is coupled to the pressure gradient along SC. As SC height decreases with increasing *r*_*je*_, the time-averaged pressure drop along SC increases (figure 4*c*). A maximum pressure drop of approximately 1 mmHg is observed for the highest value of *r*_*je*_. The peak-to-peak amplitude of the pressure oscillations in SC increases from 2.2 to 5.2 mmHg at the CC ostium as *r*_*je*_ increases (figure 4*d*).

Figure 4*e* shows that the time-averaged flow rate through SC, *q*, for any given *X* is insensitive to *r*_*je*_. This is a consequence of the inflow rate *q*_*n*_ being constant. The amplitude and phase of the oscillatory flow through SC, however, vary considerably with increasing *r*_*je*_ (figure 4*f*). Note that at no point does the flow reverse in SC (i.e., the values of *q* are always positive) with normal TM stiffness.

Figure 4*g* shows the time-averaged wall shear stress in SC, *τ*_sc_. For normal values of *r*_*je*_, the shear stress increases approximately linearly with *X*. For higher values of *r*_*je*_, *τ*_sc_ rises sharply near *X* = 1, concomitant with a decrease in SC height (figure 4*a*) and the requirement that *τ*_sc_ ∝ *q*/*h*^2^. This relationship, in conjunction with the synchronization of maximum flow and minimum SC height (figure 4*b*,*f*), causes the amplitude of the oscillatory shear stress to be highly sensitive to *r*_*je*_ (figure 4*h*). At the highest value of *r*_*je*_, which corresponds to *p*_ac_ ≈ 28 mmHg, *τ*_sc_ at *X* = 1 transiently exceeds 30 dyn cm^−2^.

Figure 4*i*,*j* show how the time-averaged TM strain, *ɛ*, varies along SC and how the instantaneous TM strain varies in time at the CC ostium. These appear as reflections of figure 4*a*,*b*, because *ɛ* is directly related to *h*. The time-averaged magnitude of *ɛ* increases with *r*_*je*_, reaching more than 20%. Notably, the oscillatory component of *ɛ* is relatively small compared to the time-averaged value.

Figure 4*k* shows the shear stress predicted to act on the endothelial cells lining the DV, *τ*_*d*_, calculated with the assumption of Poiseuille flow with an assumed uniform DV diameter of 30 *µ*m [19]. As the flow rate through the DV scales with 2*q*(1, *T*), *τ*_*d*_ follows the tracings given in figure 4*f* .

### 3.2. Sensitivity to inner wall/JCT resistance

**Figure 5.**
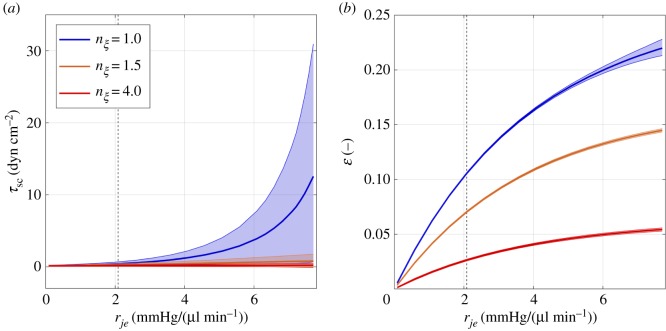
The shear stress, *τ*_sc_, on SC endothelium (*a*) and strain, *ɛ*, on TM (*b*) evaluated at the CC ostium (*X* = 1) as a function of inner wall/JCT resistance, *r*_*je*_. Colours show the effect of increasing TM stiffness by 50% (orange) and 300% (red). Solid lines show time-averaged values, and shaded regions show the oscillatory amplitude. Dashed vertical lines indicate the normal physiological value of *r*_*je*_.

The shear stress and strain acting on SC and TM cells are sensitive to inner wall/JCT resistance. The blue curves in figure 5*a*,*b* summarize the data from figure 4*h*,*j*, respectively, evaluated at the CC ostium (*X* = 1) where the time-averaged values are greatest. The central solid curves in figure 5 indicate the time-averaged values, while the shaded regions represent the amplitude of the oscillations.

For the case of normal TM stiffness (*n*_*ξ*_ = 1) shown in blue, the mean and oscillatory components for *τ*_sc_ increase sharply with *r*_*je*_ (figure 5*a*). At the normal physiological value of *r*_*je*_ (indicated by the vertical dashed line in figure 5*a*), the time-averaged shear stress is 0.4 dyn cm^−2^ and the peak-to-peak amplitude is 0.5 dyn cm^−2^. At the highest examined value of *r*_*je*_ (a glaucomatous level), the time-averaged shear stress is 29-fold greater and the peak-to-peak amplitude is 57-fold greater than for normal *r*_*je*_. At the highest examined value of *r*_*je*_, *τ*_sc_ is highly oscillatory with a peak-to-peak amplitude that is 241% of its time-averaged value. Thus, for normal values of TM stiffness, both the steady and transient components of the SC shear stress are exquisitely sensitive to increasing *r*_*je*_ and corresponding elevations in IOP. The sensitivity of *τ*_sc_ is greater towards the upper range of *r*_*je*_.

Likewise, TM strain also increases with inner wall/JCT resistance (figure 5*b*). For *n*_*ξ*_ = 1, the maximum time-averaged value of *ɛ* is approximately twice the value predicted for normal *r*_*je*_ (for which *ɛ* = 0.11). Relative changes in *ɛ* to changes in *r*_*je*_ are greater towards the lower range of *r*_*je*_. The oscillation amplitude of *ɛ* increases by a factor of 9 from an amplitude of 0.002 at physiological *r*_*je*_, but remains relatively small compared to *τ*_sc_, reaching only 7% of its time-averaged value at the highest examined value of *r*_*je*_. Thus, despite the ocular pulse, our model predicts that oscillatory strain on TM cells is unlikely to have a major role in mechanosensation within the conventional outflow pathway.

**Figure 6.**
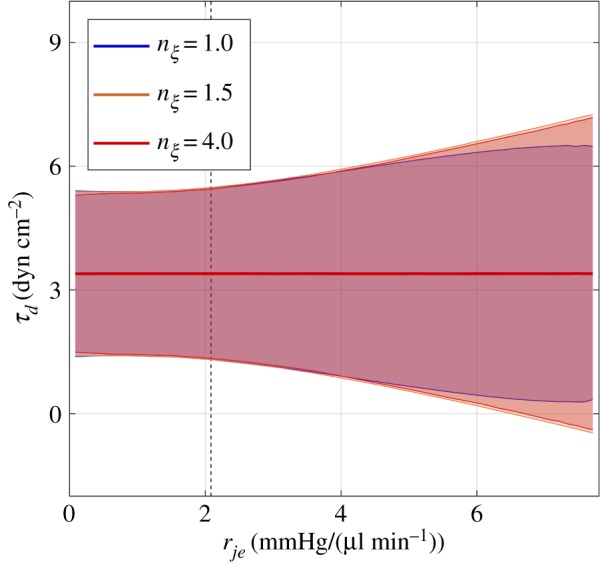
The shear stress on the endothelium of the distal vessels as a function of inner wall/JCT resistance, *r*_*je*_. Data are estimated based on the assumption of 30 collector channels, each with a diameter of 30 *µ*m. Colours show the effect of increasing TM stiffness by 50% (orange) and 300% (red). Solid lines show time-averaged values, and shaded regions show the oscillatory amplitude. Dashed vertical line indicates the ‘normal’ physiological value of *r*_*je*_.

For the DV shear stress (figure 6), the mean value of 3.4 dyn cm^−2^ does not change as *r*_*je*_ increases. This is because the net flow through these vessels and their dimensions remain constant in the model. However, the oscillatory component shows a moderate increase of 50%, from a peak-to-peak amplitude of 4.2 dyn cm^−2^, as *r*_*je*_ increases from its ‘normal’ physiological value.

### 3.3. Role of TM stiffness

Increasing TM stiffness suppresses the influence of *r*_*je*_ on SC shear stress and TM strain (figure 5*a*,*b*), and thereby decreases the sensitivity of these variables to inner wall/JCT resistance and IOP. For comparison purposes, we herein define sensitivity as the ratio of the shear stress or strain at the maximum *r*_*je*_ to that at physiological *r*_*je*_.

For *τ*_sc_, the sensitivity of both the time-averaged and oscillatory components are reduced by 90% in response to a 1.5-fold (*n*_*ξ*_ = 1.5) and 96% in response to a fourfold (*n*_*ξ*_ = 4) increase in TM stiffness. The time-averaged shear stress at the maximum examined value of *r*_*je*_ is reduced to 6% and 2% relative to the physiological case for *n*_*ξ*_ = 1.5 and *n*_*ξ*_ = 4, respectively, while the peak-to-peak amplitude is reduced by more than 99.9% for both cases. Hence, increased TM stiffness almost entirely eliminates the influence of *r*_*je*_ on SC shear stress.

For TM strain, the sensitivity of the oscillatory components of TM strain is reduced by 79% for both *n*_*ξ*_ = 1.5 and 4, but this amplitude was already negligibly small (<0.02) for the case where *n*_*ξ*_ = 1. Increasing TM stiffness decreases the maximum value of *ɛ* by 33% and 75% for *n*_*ξ*_ = 1.5 and 4, respectively. However, increasing TM stiffness has a negligible impact on the sensitivity of the time-averaged TM strain to changes in *r*_*je*_. This is because increasing *n*_*ξ*_ preserves the general form of the relationship between *ɛ* and *r*_*je*_ (figure 5*b*).

For the DV shear stress, increasing the TM stiffness has no influence on the average value, but acts to slightly increase the amplitude of oscillatory component at elevated values of *r*_*je*_.

## 4. Discussion

### 4.1. Physiological background

SC is a collapsible endothelial-lined vessel that is tethered to the underlying JCT and TM [20,21]. Increasing IOP tends to narrow the lumen of SC and correspondingly stretch the tissues of the TM [22–25]. This generates at least two distinct mechanical cues acting on TM and SC cells in response to IOP elevation: (i) stretch on TM cells as the TM/JCT expands [8]; and (ii) shear stress on SC endothelial cells, due to circumferential flow through a narrowing SC lumen [10,26]. We developed a mathematical model to estimate the magnitude of these mechanical forces in human eyes. The model predicts that SC shear stress and TM strain are highly sensitive to inner wall/JCT resistance. This suggests that both TM strain and SC shear stress act as IOP-dependent mechanical cues to trigger homeostatic mechanisms for IOP regulation within the conventional outflow pathway. While each of these mechanisms has been identified individually [8–11,26–28], the current work implies for the first time a complementarity between these two mechanical cues through sensitivity to different ranges of inner wall/JCT resistance.

Experimental evidence shows that conventional outflow resistance is actively regulated for IOP homeostasis. Bradley *et al.* [8] demonstrated that in response to doubling the flow rate in an organ culture perfusion system, IOP initially doubled but then returned towards baseline values over several days. Elevated MMP-2 (gelatinase A) activity over this period suggested that ECM remodelling was involved in the reduction in outflow resistance responsible for IOP normalization [8]. The authors concluded that this mechanism was likely mediated via TM strain, because stretch applied to cultured TM cells induced similar increases in MMP-2 activity [8]. Comparable changes in outflow resistance in response to prolonged IOP elevation were reported by other investigators [29], and recent studies suggest that this regulatory mechanism may be disrupted in glaucoma [18]. Thus, a homeostatic mechanism for IOP regulation appears to function within the conventional outflow pathway, and its disruption may contribute to the pathogenesis of ocular hypertension in glaucoma.

Any potential mechanism for IOP homeostasis requires a means to sense the magnitude of IOP itself. Based on the observation of adaptive responses in the conventional outflow pathway [8,29], we postulate that mechanosensation of IOP likely occurs within the TM and SC. These tissues are also responsible for the bulk of outflow resistance generation [4,5]. This places the putative sensors and effectors of IOP within the same anatomical location, allowing a *local* response to perturbations in inner wall/JCT resistance. The purpose of this study was to elucidate the magnitudes and characteristics of the mechanical cues involved in IOP mechanosensation in the TM and SC and to examine how elevated TM stiffness may disrupt these cues.

### 4.2. Model predictions

This section summarizes and contextualizes the four main predictions from the model.

#### 4.2.1. Model prediction 1: magnitudes of TM strain and SC shear stress

Under normal physiological conditions, the model predicts that TM strain is approximately steady with 11% extension, while SC shear stress is rather small (<0.7 dyn cm^−2^). With increasing inner wall/JCT resistance and IOP, TM strain increases moderately by up to a factor of 2, while SC shear stress exhibits a sharp increase and becomes highly oscillatory, reaching a time-averaged value of 11 dyn cm^−2^ with a peak-to-peak amplitude of 28 dyn cm^−2^ near the CC ostium.

The predicted values of TM strain under *normal* conditions and SC shear stress under *hypertensive* conditions should both be sufficient to stimulate the release of resistance-modulating compounds based on *in vitro* studies of TM and SC cells. For example, static or oscillatory (1 Hz) mechanical stretch of TM cells at 5 or 10% stimulates MMP-2, MMP-9 and transforming growth factor beta-1 (TGF-*β*1) that contribute to ECM synthesis and turnover [8,27,30,31]. Oscillatory stretch applied to TM cells stimulates release of ATP [32], adenosine [9] and VEGF [11], all of which may influence the permeability of the inner wall endothelium of SC. A steady shear stress of 10 dyn cm^−2^ induces NO production by SC cells [33], which lowers outflow resistance [10,28,34–36].

The predicted magnitudes of shear stress and strain provide a range to further sample the mechanobiological responses of SC and TM cells to shear and stretch *in vitro*. As SC cells are vascular derived, and vascular endothelial cells are sensitive to oscillatory shear stress [37–39], it would be beneficial for future shear stress studies of SC cells to investigate oscillatory flow to complement previous studies using steady shear stress [33]. The predicted magnitudes indicated in figure 5*a* provide an appropriate range to investigate the SC shear response *in vitro*. Regarding TM cells, our predictions suggest that stretch studies should use static or quasi-static stretch where possible, as the ocular pulse appears unlikely to add significant oscillatory strain to TM cells. Interpreting the magnitude of strain acting on TM cells is more complicated.

For the TM and JCT, there is a question of how tissue strain translates into a mechanical stimulus experienced by individual TM cells. This depends on the details of the local tissue architecture and how TM cells are adhered to their surrounding ECM and neighbouring cells. For example, strain experienced by a TM cell adhered to a relatively rigid corneoscleral beam may be very different from that experienced by a JCT cell embedded within loose ECM and having numerous contacts to neighbouring cells. Additionally, time-dependent remodelling of ECM and cellular adhesions within the TM in response to prolonged stretch may also significantly affect the strain magnitude experienced by TM cells. Furthermore, the way in which the TM is tethered to surrounding tissue may affect its response to applied loads. For example, if the TM bends like a beam [40], rather than expanding like a spring, then the absolute value of the TM strain would be reduced. The combined effect of these uncertainties and current limitations on imaging the TM make it difficult to predict the absolute strain experienced by TM cells. As a first-order approximation, it may be reasonable to assume that TM remodelling in healthy tissue at the ‘normal’ physiological state would offset cellular strain values to zero. From this baseline state, steady tensile and compressive strains ranging from ± 10% would be appropriate for *in vitro* studies.

#### 4.2.2. Model prediction 2: differential sensitivities of TM strain and SC shear stress

The model predicts that TM strain and SC shear stress are sensitive to different ranges of inner wall/JCT resistance. TM strain is more sensitive to perturbations in resistance around the normal physiological state, while SC shear stress is more sensitive to perturbations when inner wall/JCT resistance is elevated several fold above normal. This suggests that the conventional outflow pathway may exploit multiple mechanosensory mechanisms that are each tuned to different ranges of outflow resistance. For example, small perturbations around the normal setpoint may induce slow responses via TM strain, such as MMP remodelling that operates over the timescale of days [8], while large perturbations may induce fast responses via SC shear stress, such as NO production that immediately decreases outflow resistance and IOP [10,28,34–36]. Thus, the combination between TM strain and SC shear stress may allow the outflow pathway to differentiate and respond appropriately to acute versus chronic perturbations in outflow function, providing robust mechanosensation and IOP homeostasis.

#### 4.2.3. Model prediction 3: effect of TM stiffness

TM stiffness appears to be a key regulator of mechanosensation within the conventional outflow pathway. Increasing TM stiffness, even by a modest 50%, desensitizes SC shear stress and TM strain to perturbations in inner wall/JCT resistance. Much larger changes in TM stiffness may be observed in glaucoma, with Last *et al.* [12] reporting a 20-fold increase in TM stiffness and Wang *et al.* [13] reporting a twofold increase, although the latter did not achieve statistical significance. A stiffer TM renders the outflow pathway less sensitive to mechanosensory cues that may otherwise trigger feedback signals for IOP homeostasis. Elevated TM stiffness may thereby contribute to the pathogenesis of ocular hypertension and glaucoma by impairing the ability of the outflow pathway to sense and respond to incremental changes in outflow resistance and IOP. This could explain the reported loss of IOP homeostasis in post-mortem glaucomatous eyes [18]. Conversely, reducing TM stiffness or the contractility of TM cells (for example using rho kinase inhibitors) may increase outflow facility by increasing shear stress in SC and promoting shear-induced homeostatic mechanisms.

#### 4.2.4. Model prediction 4: mechanosensation in distal vessels

The endothelial cells lining the CCs originate from vascular endothelial cells [41], which are sensitive to shear stress by secreting NO and other shear-induced signals. NO donors have been shown to decrease distal resistance, presumably by acting on surrounding smooth muscle cells [42] to increase the calibre of DV [43]. Conversely, the physiological antagonist to NO, endothelin-1, increases distal resistance [43]. This implies that the DV are capable of responding to changes in shear stress, but not necessarily changes in IOP.

To the best of our knowledge, the only evidence showing changes in distal resistance with IOP was generated in enucleated eyes using a constant pressure perfusion technique [6]. However, elevations in IOP with this technique coincide with increased flow rate that would impose artificially elevated shear stresses in the DV. It is therefore possible that the decrease in distal resistance reported with increasing IOP [6] could have been mediated via artificially elevated shear stress in the DV that led to NO-driven vasodilation. Such a response would not occur *in vivo* because aqueous humour flow is approximately constant.

Correspondingly, the model predicts that the steady component of the shear stress in the DV is relatively independent of inner wall/JCT resistance. The modest 50% increase in the amplitude of the oscillatory component of the shear stress is small compared to the magnitude of the changes predicted in SC. Thus, unless the endothelial cells lining the DV are extremely sensitive to changes in oscillatory shear stress, the implication from the model is that the shear stress in the DV does not play a major role in mechanosensation of IOP elevations. However, this analysis does not preclude a role for active processes such as innervation that may control the tone of CC, intrascleral vessels or arteriovenous anastomoses [44].

### 4.3. Model strengths and limitations

In the formulation of the model, we aimed to capture the critical aspects of the outflow pathway in the simplest possible manner. Building on a previous study [7], the model added the ocular pulse, the constant net flow condition, the non-linearly collapsible TM, the resistance of the DV and oscillatory EVP. We made a number of assumptions regarding the model geometry and the parameters.

#### 4.3.1. Model geometry

The model assumes an idealized SC geometry represented by a high aspect ratio rectangular channel. This neglects the true *in vivo* shape of SC, which may better be considered elliptical in cross-section [26] but is in practice signficantly more complicated and occasionally bifurcated by septa into multiple lumina [45]. Septa may also limit the change in SC height with increasing IOP [7]. The model considers only a small portion of SC, equivalent to 1/2*n* of its total circumference and assumes that each of the 2*n* segments behaves identically and occupy a uniform half-width *x*_0_ between CC ostia. Non-uniform or segmental outflow, as occurs in human eyes [18,46], will introduce regional variability in the quantity of flow passing through the TM and SC. The presence of an endothelial glycocalyx [47] may also influence the fluid mechanics near the SC cell surface or the cellular response to shear stress. These complexities will cause the true *in vivo* values of TM strain and SC shear stress to deviate quantitatively from the model predictions. However, as the model was based on typical anatomical parameters, the predictions should provide reasonable estimates of the characteristic values of strain and shear stress experienced by TM and SC cells *in vivo*. More sophisticated models that incorporate the micro-anatomy of TM and SC could provide more detailed estimates of TM strain and SC shear stress but would be subject to additional modelling complexity and uncertainty.

The present model qualitatively predicts how TM strain and SC shear stress are sensitive to different ranges of inner wall/JCT resistance and how TM stiffness disrupts this mechanosensitivity. These qualitative predictions should be relatively independent of anatomical variability in the outflow pathway between individuals. This is because the anatomy is relatively constant for a given individual, as would be the effect of the anatomy on the aqueous filtration pathways through the TM and SC. This is supported by tracer studies showing that, despite significant variability in tracer patterns between individuals, segmental outflow pathways through the TM are relatively insensitive to changes in IOP within individual human eyes [46].

We calculated the role of shear stress on the endothelial cells lining DV by assuming that the diameter was uniform and equal to 30 *μ*m, based on histology studies in humans [19,45]. This neglects any constrictions or choke-points in the DV, where shear stress may be locally elevated. While the shear stress averaged over the length of the DV is likely to be small (otherwise distal resistance would be larger), the shear stress may take on larger values at discrete locations to trigger a local shear-induced response. However, without knowing the morphology and how vessel calibre changes with IOP, it is not possible to make more definitive conclusions on the role of shear stress in the DV.

#### 4.3.2. Parameter sensitivity

Although the model contains a large number of input parameters (16 fixed and one independent), all of these are either set based on literature values or calculated based upon fixed values. In our analysis, we considered only one independent parameter, the inner wall/JCT resistance *r*_*je*_ and one investigated parameter *n*_*ξ*_ representing the relative increase in TM stiffness. The quantitative predictions of the model will be somewhat sensitive to particular values or uncertainties in the parameter estimates. However, for any given set of parameters, the TM strain and SC shear stress will still depend on *r*_*je*_ and *n*_*ξ*_, and this dependence will be qualitatively similar to the predictions of the model.

TM stiffness was found to be a critical parameter influencing TM strain and SC shear stress. Rather than choosing a particular value of TM elastic modulus, which varies broadly in the literature (0.1 kPa–50 MPa) [17], we took an empirical approach to relate SC height to the pressure drop across the inner wall. This approach captures the key relationship between IOP and SC height independent of boundary conditions or exact values of elastic modulus. While this approach yielded an effective value of TM reference apparent stiffness *ξ*_0_, this parameter is not directly comparable to the TM elastic modulus, as might be determined experimentally. However, multiplicative changes in TM stiffness by varying *n*_*ξ*_ would have the same effect as a proportional change in the elastic modulus.

Distal resistance *r*_*d*_ was defined by assuming that 25% of the total outflow resistance under normal physiological conditions resides in the DV [48]. This value of *r*_*d*_ was then assumed to be constant for all values of inner wall/JCT resistance and IOP. While it has been reported that distal resistance decreases with increasing IOP [6], the exact form of the relationship between *r*_*d*_ and IOP remains unknown. Furthermore, as discussed above, it remains unclear whether the reported change in distal resistance is attributable to IOP itself or rather to concomitant elevations in distal shear stress due to the experimental technique. Furthermore, for any given IOP (which for a constant flow system implies a unique total resistance), decreasing *r*_*d*_ would increase the pressure drop across the inner wall and thereby increase the TM strain and SC shear stress. Therefore, if the model had included decreasing *r*_*d*_ with increasing IOP, this would have enhanced the sensitivity to IOP elevation, although not by a significant amount. We therefore opted to keep *r*_*d*_ constant in our simulations for simplicity.

## 5. Conclusion

In conclusion, this study reveals that SC shear stress and TM strain may provide complementary mechanosensory cues for homeostatic regulation of outflow resistance and hence IOP. The estimated magnitudes of TM strain and SC shear stress are sufficient, based on prior *in vitro* studies, to trigger the release of NO, VEGF, MMPs or other resistance-modulating compounds that may serve as feedback signals for IOP homeostasis. TM strain and SC shear stress are sensitive to different ranges of inner wall/JCT resistance, allowing the outflow pathway to differentially sense small versus large perturbations in IOP. Elevated TM stiffness, as occurs in glaucoma, is predicted to impair IOP homeostasis by decoupling outflow resistance and IOP from TM strain and SC shear stress. This suggests a novel mechanism to explain how elevated TM stiffness may be causative for the pathogenesis of ocular hypertension and glaucoma.

## Supplementary Material

Appendix: Mathematical Model Formulation

## Supplementary Material

Matlab Code

## Data accessibility

Code for generating data has been included in the supplementary MatlabCode.rar file.
