# Supplementary material for: A model of the oscillatory mechanical forces in the conventional outflow pathway
Source: J R Soc Interface. 2019 Jan 30;16(150):20180652. doi: 10.1098/rsif.2018.0652 (PMC6364644; doi:10.1098/rsif.2018.0652)
Supplement: Appendix: Mathematical Model Formulation [file rsif20180652supp1.pdf]

Oscillatory Mechanical Forces in the Conventional Outflow Pathway
Appendix: Model Formulation

Joseph M. Sherwood, W. Daniel Stamer, Darryl R. Overby

The model used in this study builds on the one dimensional TM/SC model of Johnson and Kamm [1]. Here, we make four major modifications: i) we couple the TM/SC model to lumped parameter models representing the rest of the eye; ii) we consider an '*in vivo*' paradigm, in which the net flow through the conventional outflow pathway is constant; iii) we consider the oscillatory nature of the flow and resulting mechanical forces in the conventional outflow pathway due to the ocular pulse; and iv) we account for the non-linear pressure-height relationship of Schlemm's canal.

A1 Lumped Parameter Model of the Whole Eye

In order to model the interaction between the TM/SC and the rest of the eye, we couple the one dimensional TM/SC model to the lumped parameter model shown in Figure A1a. This model comprises the corneoscleral globe and anterior chamber, the conventional outflow pathway, and the downstream vasculature.

Upstream of the TM/SC, there is: a steady flow source q_n, representing the net pressure-independent aqueous humour flow (the difference between the rate of aqueous humour secretion and any pressure-independent outflow); an oscillatory flow source q_b, representing the flow of blood into and out of the intraocular vasculature during the cardiac cycle (which has zero mean); and the pressure-dependent compliance of the corneoscleral globe ϕ_g. Downstream of the TM/SC, r_d represents the hydrodynamic resistance of the distal vessels that allow flow from SC to the episcleral vessels.

The episcleral vessel pressure (EVP), notated $p_{ev}(t)$, is modelled as an oscillatory pressure source, following studies showing the oscillatory nature of EVP [2, 3]. This model is appropriate if the oscillations in EVP are insensitive to the aqueous humour flow rate entering the episcleral vessels, an assumption made on the basis that the net blood flow rate within the episcleral vessels is expected to be orders of magnitude greater than the aqueous humour flow rate.

The episcleral vessel pressure is modelled as a sinusoid at the frequency of the ocular pulse, f, and with a phase lag relative to the ocular pulse of ψ_{ev}, based on observations of servo-null measurements in a rabbit [2, 3]:

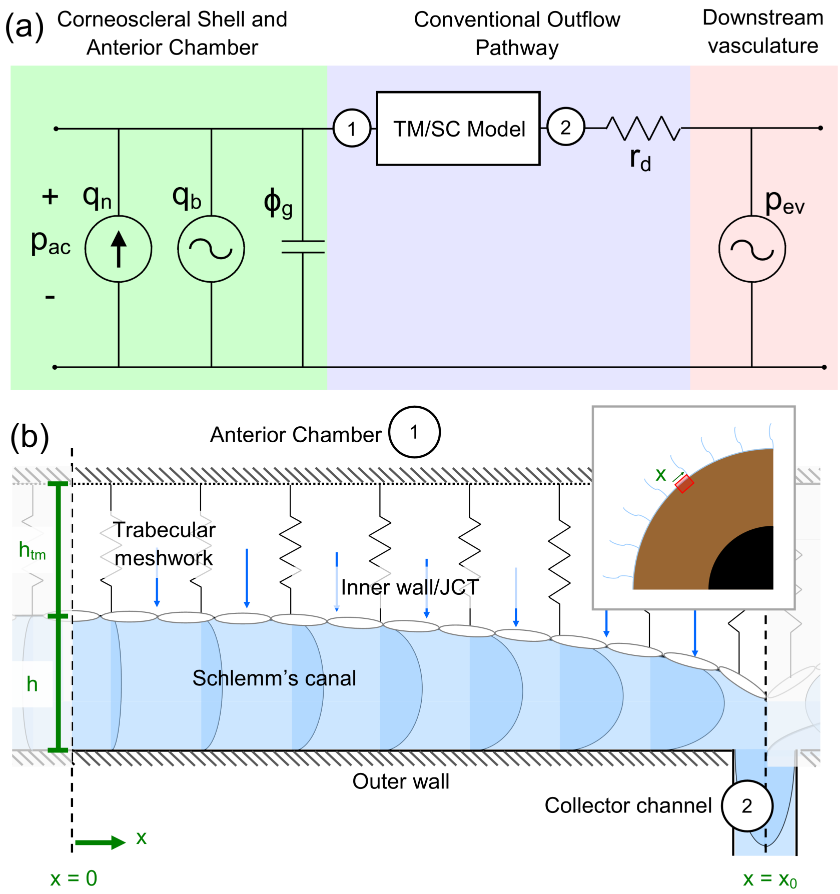

Figure A1: (a) Coupling the TM/SC model to a lumped parameter model of the whole eye. Circled numbers represent the locations where the TM/SC model interfaces at the anterior chamber ① and collector channels ②. (b) A schematic of an individual segment of the TM/SC model. Inset: frontal view of the eye indicating the mathematical domain (red rectangle) relative to collector channels (blue), iris (brown) and pupil (black).

$$p_{ev}(t) = \overline{p_{ev}} + a_{ev} \sin(2\pi f t - \psi_{ev}) \tag{1}$$

where $\overline{p_{ev}}$ is the average EVP and a_{ev} is the EVP oscillation amplitude.

Similarly, the anterior chamber pressure can be decomposed into steady and unsteady components, such that

$$p_{ac}(t) = \overline{p_{ac}} + p'_{ac}(t) \tag{2}$$

The steady component of the AC pressure, $\overline{p_{ac}}$, is given by the sum of $\overline{p_{ev}}$ and the pressure drop arising from flow of aqueous humour across the resistance of the conventional outflow pathway. This resistance includes

r_{je} (the combined resistance of the SC inner wall and JCT) and r_d (the resistance of the distal vessels), as well as the resistance arising from viscous losses in SC. Although the resistance of SC varies as a function of time as SC height changes, the overall effect on $\overline{p_{ac}}$ can be represented by an apparent SC resistance, $\widetilde{r_{sc}}$. The value of $\widetilde{r_{sc}}$ is the resistance that would result in the same elevation of $\overline{p_{ac}}$ under steady conditions. The value of $\overline{p_{ac}}$ is therefore given by

$$\overline{p_{ac}} = q_n \left(r_{je} + r_d + \widetilde{r_{sc}}\right) + \overline{p_{ev}} \tag{3}$$

The oscillatory term $p'_{ac}(t)$, can be calculated based on the flow of blood into the compliance of the corneoscleral globe:

$$q_b(t) = \phi_g \frac{dp_{ac}(t)}{dt} \tag{4}$$

For the present model, we let $q_b(t) = a_{qb} \cos(2\pi f t)$, where a_{qb} is the amplitude of the blood flow entering the eye during the ocular pulse at frequency f. Integrating Equation 4 (with the assumption that the pressure oscillations are sufficiently small that the compliance of the corneoscleral globe does not vary over the cardiac cycle) yields

$$p'_{ac}(t) = \frac{1}{\phi_g} \int_0^t a_{qb} \cos(2\pi f t)\, dt = a_{ac} \sin(2\pi f t) \tag{5}$$

where the amplitude of the pressure oscillations in the anterior chamber (the ocular pulse amplitude) is given by

$$a_{ac} = \frac{a_{qb}\, k\, \overline{p_{ac}}}{2\pi f} \tag{6}$$

where the ocular compliance is estimated using Friedenwald's coefficient of ocular rigidity, k, according to $\phi_g = 1/(p_{ac}\, k)$.

A2 Model of the TM and SC

A schematic of a section of SC, based on that of Johnson and Kamm [1], is given in Figure A1b. The section is defined between $x = 0$ at a point equidistant between collector channels, and $x = x_0$ at the collector channel ostium. The height of the TM is $h_{tm}(x, t)$, and the canal height $h(x, t)$ is determined

by the apparent stiffness of the TM, and the pressures in the anterior chamber, $p_{ac}(t)$, and SC, $p_{sc}(x,t)$. Fluid crosses the inner wall and flows along SC to the collector channel (CC). There are $2n$ such segments in parallel making up the complete SC, where n is the number of collector channels. The inset shows the channel section dimensions, with width w.

A2.1 Fundamental Equations

The filtration velocity across the inner wall, $u_{iw}(x,t)$, is given by

$$u_{iw}(x,t) = \frac{1}{2nwx_0 r_{je}} \left(p_{ac}(t) - p_{sc}(x,t)\right) \tag{7}$$

where $2nwx_0$ is the en face area of the entire SC inner wall and r_{je} is the total hydrodynamic resistance of the full circumference of the SC inner wall and JCT.

Conservation of mass within the canal yields

$$\frac{\partial q(x,t)}{\partial x} = \left(u_{iw}(x,t) - \frac{\partial h(x,t)}{\partial t}\right) w \tag{8}$$

where $q(x,t)$ is the flow rate in SC in the direction towards the SC ostium. Note that Equation 8 is valid when $(dh/dx)^2 \ll 1$. For the present study, the largest $(dh/dx)^2$ was 0.0009, so this assumption is appropriate.

Due to the tethering between the trabecular meshwork and inner wall of SC [4, 5], we assume that the total height of the TM and SC together does not vary (hence $h + h_{tm}$ is constant). Hence, we can write

$$p_{ac}(t) - p_{sc}(x,t) = \xi \left(\frac{h_0 - h(x,t)}{h_0}\right) \tag{9}$$

where ξ is an apparent stiffness that characterises the pressure-dependent changes in SC height and h_0 is the resting SC height in the absence of a pressure drop across the inner wall.

The pressure drop due to viscous losses along SC is estimated based on the analytical solution for pressure-driven flow between two flat plates:

$$\frac{\partial p_{sc}(x,t)}{\partial x} = -\frac{2nr_{sc,0}h_0^3}{x_0}\frac{q(x,t)}{h^3(x,t)} \tag{10}$$

The parameter $r_{sc,0}$ is the hydrodynamic resistance of $2n$ parallel SC segments of length x_0 at $h = h_0$, estimated using the aforementioned analytical solution for a fluid with viscosity μ:

$$r_{sc,0} = \frac{12\mu x_0}{2nwh_0^3} \tag{11}$$

The use of the assumption of flow between two flat plates for Equations 10 and 11 requires several assumptions: i) that inertial effects are negligible compared to viscous effects, as both Womersley and Reynolds numbers are significantly less than 1, ii) that $w \gg h$, such that the shear force on the anterior and posterior sides of SC can be neglected, and iii) that $h_0 \ll x_0$, such that the pressure gradient and velocity in the transverse direction are negligible. For this latter assumption, the current parameters yield $\frac{h_0}{x_0} = 0.04$, hence the assumption is valid.

A2.2 Boundary and Initial Conditions

Boundary Condition at $x = 0$: this is a plane of symmetry, hence

$$\frac{\partial h\left(0,t\right)}{\partial x} = 0 \tag{12}$$

Boundary Condition at $x = x_0$: the SC pressure at the CC ostium is the sum of $p_{ev}\left(t\right)$ and the product of the flow through SC and the distal resistance:

$$p_{sc}\left(x_0,t\right) = p_{ev}\left(t\right) + 2nr_d\, q\left(x_0,t\right) \tag{13}$$

where r_d is the hydrodynamic resistance of the distal portions of the outflow pathway comprising all collector channels and intrascleral vessels.

Initial Condition at $t = 0$: we select the initial condition as the steady state solution for the special case that $\widetilde{r_{sc}} = 0$:

$$h_s = h_0\left(1 - \left(\frac{\overline{p_{ac}} - \overline{p_{ev}}}{\xi}\right)\left(\frac{r_{je}}{r_d + r_{je}}\right)\right) \tag{14}$$

A2.3 Non-Dimensionalisation

In order to simplify the equations, we non-dimensionalise using the characteristic scales outlined in Table A1. Dimensionless variables are given in capital Roman letters and dimensionless parameters are given by Greek letters (except ξ, ϕ_{sc} and ϕ_g).

Table A1: Dimensionless Variables and Parameters

Definition	Description
$H = \frac{h}{h_0}$	Dimensionless canal height
$X = \frac{x}{x_0}$	Dimensionless SC location
$P = \frac{p}{\xi},\ A = \frac{a}{\xi}$	Dimensionless pressures
$T = ft$	Dimensionless time
$\phi_{sc} = \frac{2nwx_0h_0}{\xi}$	Compliance of SC
$\alpha = \phi_{sc} r_{je} f$	Ratio of time scales of TM relaxation and ocular pulse
$\beta = \frac{r_{je}}{r_{sc,0}}$	Ratio of inner wall/JCT resistance to reference SC resistance
$\zeta = \frac{r_{je}}{r_d}$	Ratio of inner wall/JCT resistance to distal resistance
$\Pi = \frac{p_{ac} - p_{ev}}{\xi}$	Dimensionless pressure drop across conventional outflow pathway
$\Delta = \sqrt{A_{ac}^2 + A_{ev}^2 - 2A_{ac}A_{ev}\cos(\psi_{ev})}$	Dimensionless amplitude of $P_{ac}(T) - P_{ev}(T)$
$\psi_{sc} = \tan^{-1}\left(\frac{A_{ev}\sin(\psi_{ev})}{A_{ac} - A_{ev}\cos(\psi_{ev})}\right)$	Phase lag of $P_{ac}(T) - P_{ev}(T)$

Governing Equation: combining Equations 7-10 and non-dimensionalising yields

$$H(X,T) - 1 + \alpha\left(\frac{\partial H(X,T)}{\partial T}\right) = \frac{\beta}{4}\left(\frac{\partial^2 H^4(X,T)}{\partial X^2}\right) \tag{15}$$

Boundary Condition at $X = 0$: Equation 12 becomes

$$\frac{\partial H(0,T)}{\partial X} = 0 \tag{16}$$

Boundary Condition at $X = 1$: combining Equations 9, 10 and 13 and non-dimensionalising yields

$$P_{ac}(T) - P_{ev}(T) = 1 - H(1,T) - \frac{\beta}{4\zeta}\frac{\partial H^4(1,T)}{\partial X} \tag{17}$$

The coupling between the lumped parameter and TM/SC models appears through the left hand side of Equation 17. Combining Equations 1, 2, 5 and 17, simplifying with trigonometric identities and non-dimensionalising, we can write the boundary condition at $X = 1$ as

$$\frac{\partial H^4(1,T)}{\partial X} = -\frac{4\zeta}{\beta}\left(\Pi + \Delta \sin\left(2\pi T - \psi_{sc}\right) + H(1,T) - 1\right) \tag{18}$$

Initial Condition at $T = 0$: non-dimensionalising Equation 14 yields

$$H_s = 1 - \Pi\left(\frac{\zeta}{1+\zeta}\right) \tag{19}$$

Equations 15, 16, 18 and 19 can be solved numerically to yield $H(X,T)$, as described in Sections A3 - A5.

A2.4 Extracting Results

To present the results, we re-dimensionalise the main parameters, but keep X and T in dimensionless form for simplicity. The dimensional form of h is given by $h(X,T) = h_0 H(X,T)$. The dimensional SC pressure can then be calculated using Equation 9, $p_{ac}(T)$ and the final value of ξ (see §A4.7). Other key output parameters are calculated as follows.

Flow Rate: the dimensionless flow rate through a given SC section is given by

$$Q(X,T) = -\frac{\beta}{\alpha} H^3(X,T)\frac{\partial H(X,T)}{\partial X} \tag{20}$$

and the dimensional flow rate is given by

$$q(X,T) = fwh_0x_0Q(X,T) = q_0Q(X,T) \tag{21}$$

where $q_0 = fwh_0x_0$ is the flow rate required to turnover one section of SC (of which there are $2n$) completely in one cardiac cycle when $h = h_0$. Note that the flow across r_d at time T is $2nq(1,T)$.

Shear stress in SC: the dimensionless shear stress in Schlemm's canal is given by

$$\mathcal{T}_{sc}(X,T) = -\frac{\beta}{\alpha} H(X,T)\frac{\partial H(X,T)}{\partial X} \tag{22}$$

and the dimensional shear stress is given by

$$\tau_{sc}(X,T) = \left(\frac{6\mu q_0}{wh_0^2}\right)\mathcal{T}_{sc}(X,T) = \tau_{sc,0}\mathcal{T}_{sc}(X,T) \tag{23}$$

where $\tau_{sc,0} = 6\mu q_0/wh_0^2$ is the shear stress generated at the flow rate q_0 and height h_0, under the assumption of quasi-steady flow between two flat parallel plates.

TM strain: the TM strain can be calculated directly from the dimensional SC height according to

$$\varepsilon(X,T) = \frac{h_0 - h(X,T)}{h_{tm,0}} \tag{24}$$

where $h_{tm,0}$ is the TM height when $h = h_0$.

Shear stress in distal vessels: we estimate this by assuming Poiseuille flow (with a flow rate of $2q(1,T)$), hence

$$\tau_d(T) = \frac{64\mu q(1,T)}{\pi d_d^3} \tag{25}$$

where d_d is the diameter of the distal vessels.

A3 Model Coupling

While the physiological system, and the lumped parameter model describing it (Figure A1a), have q_n as an input, the dimensionless parameters in Equations 15, 16, 18 and 19 are functions of p_{ac}. Solving these Equations yields q_n as a result, but the same value of q_n given in Table A2 is required for all simulations. This necessitates an approach to identify the value of $\overline{p_{ac}}$ that yields a flow rate through the system equalling q_n. As $\widetilde{r_{sc}}$ is unknown, it is not possible to calculate $\overline{p_{ac}}$ *a priori*, and hence an iterative approach is required.

The iteration procedure is as follows, using j to indicate iteration number. An initial estimate of $\widetilde{r_{sc}}$ is used to estimate an initial value of $\overline{p_{ac}^j}$ using Equation 3. The system of equations is then solved and q_n^j is calculated according to

$$q_n^j = 2n\int_0^1 q(1,T)\,dT \tag{26}$$

The next estimate for the apparent SC resistance, $\widetilde{r_{sc}^{j+1}}$, is then given by

$$\widetilde{r_{sc}^{j+1}} = \left(\frac{\overline{p_{ac}^{j}} - \overline{p_{ev}}}{q_n^j} - r_{je} - r_d \right) (1 - \lambda) + \widetilde{r_{sc}^{j}} \lambda \tag{27}$$

where λ is a relaxation factor. $\widetilde{r_{sc}^{j+1}}$ is then used to estimate a new value of $\overline{p_{ac}^{j+1}}$ and the process is repeated until $q_n - q_n^j < q_{\text{thresh}}$, where q_{thresh} is a target threshold.

A4 Parameter Selection

Due to the interdependence of the many parameters, a hierarchical scheme is necessary for parameter selection (Figure A2).

A4.1 *I.* Fixed Parameters.

These parameters are taken directly from the literature, and are listed in the top section of Table A2. These values are kept constant for all simulations.

Table A2: Dimensional parameters. Top section indicates fixed parameters used for all simulations. Middle section indicates 'normal' parameters, required for calculating derived parameters. Bottom section provides values of derived parameters. *Calculated from aqueous humour secretion at 2.4 $\mu l/min$ [6] minus pressure-independent outflow at 10% of aqueous humour secretion rate [7].

Term	Definition	Value	Unit	Source
h_0	Reference SC height	25	μm	[1]
$h_{tm,0}$	Reference TM height	100	μm	[8]
w	SC width	300	μm	[9]
x_0	Half-width between CC	600	μm	[1]
n	Number of CC	30	-	[9, 10]
μ	Aqueous humour viscosity	0.75	$mPa\,s$	[9]
$\overline{p_{ev}}$	Average EVP	9	$mmHg$	[1]
a_{ev}	EVP oscillation amplitude	1.5	$mmHg$	[2]
ψ_{ev}	Ocular pulse-EVP phase lag	0.2π	rad	[2]
f	Frequency of ocular pulse	1	Hz	[11, 12]
k	Ocular rigidity	0.0215	$1/\mu l$	[13]
q_n	Net flow rate	2.16*	$\mu l/min$	[9],[7]
$\overline{p_{ac,N}}$	Normal average IOP	15	$mmHg$	[1]
$a_{ac,N}$	Ocular pulse oscillation amplitude at $\overline{p_{ac,N}}$	1.5	$mmHg$	[11]
$r^*_{d,N}$	Ratio of distal to total outflow resistance	0.25	-	[14]
$r_{sc,0}$	Reference SC resistance at h_0	0.0024	$mmHg/(\mu l/min)$	Eq. 11
r_d	Resistance of the distal vessels	0.694	$mmHg/(\mu l/min)$	Eq. 28
a_{qb}	Amplitude of ocular blood flow oscillations	1.75	ml/min	Eq. 30
ξ_0	Effective TM stiffness at h_0	8.2	$mmHg$	Eqs. 31 32

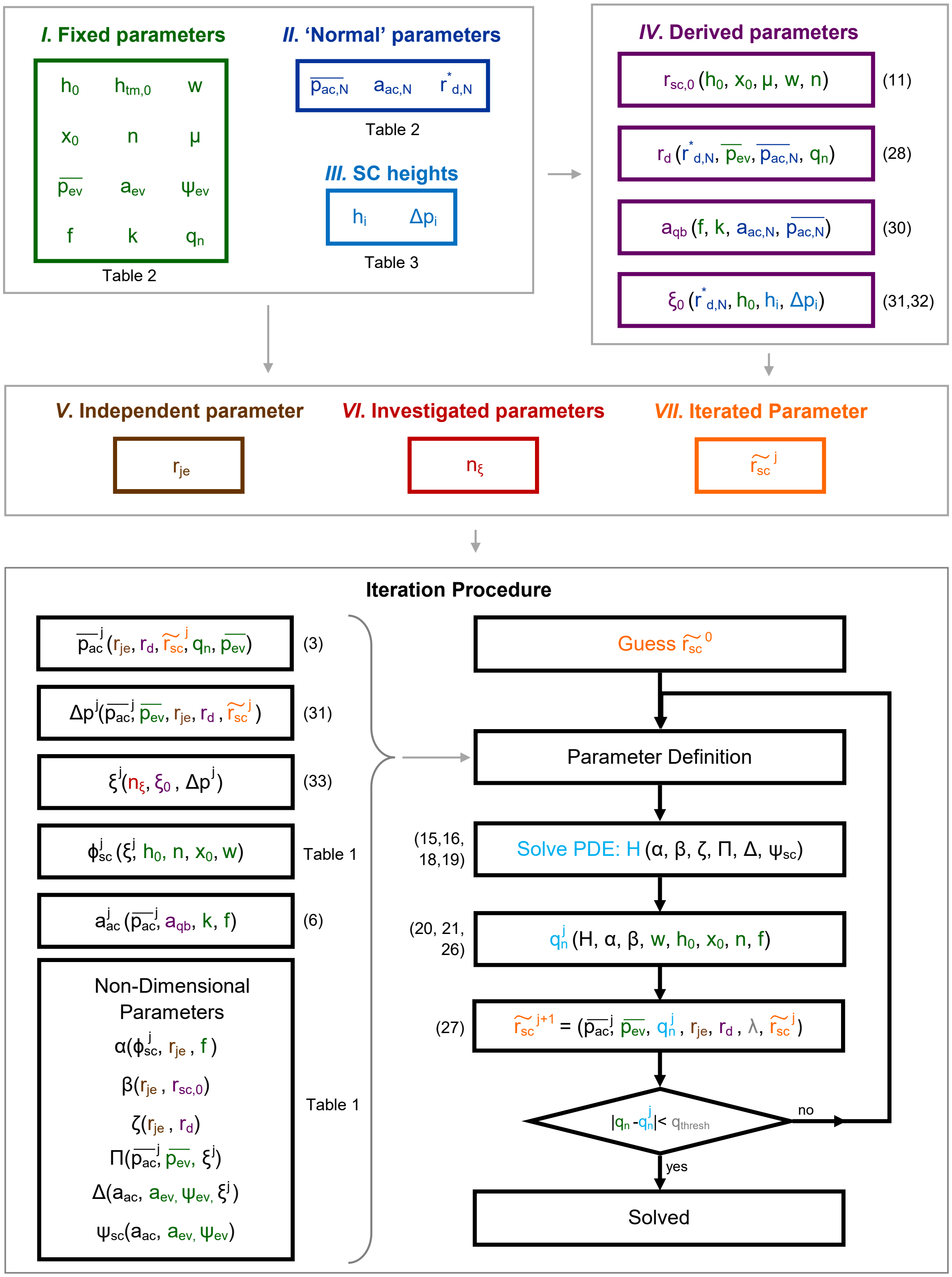

Figure A2: Outline of the iterative parameter definition and solution procedure. Numbers in parentheses refer to equations.

A4.2 *II.* 'Normal' Parameters.

The values given in the middle section of Table A2 are representative of the normotensive population. These are not used directly in the solutions, but are used to estimate the derived parameters shown in the bottom section of Table A2.

A4.3 *III.* SC heights.

In order to define the apparent stiffness ξ, we use empirical *ex vivo* data on the SC pressure-height relationship, listed in Table A3.

Table A3: Experimental values of Schlemm's canal height at different intraocular pressures. See §A4.4 for application.

$h\,(\mu m)$	$\overline{p_{ac}}\,(mmHg)$	$\Delta p\,(mmHg)$	Source
25	0	0	[1]
6.5	15	11.3	[15]
0	40	30	[16]

A4.4 *IV.* Derived Parameters

The derived parameters are calculated based on values in Table A3 and the top two sections of Tables A2, and are constant for all simulations.

SC resistance. The reference SC resistance $r_{sc,0}$ can be calculated directly from the parameters in Table A2 and Equation 11.

Distal vessel resistance. Johnson [14] concluded that the distal resistance represents approximately a quarter of the total resistance of the outflow pathway in normal eyes ($r^*_{d,N} = 0.25$). In our experimental paradigm of increasing inner wall resistance, and hence increasing total resistance, the *absolute value* of the distal resistance does not change. Hence the distal resistance is given by

$$r_d = r^*_{d,N} \left(\frac{\overline{p_{ac,N}} - \overline{p_{ev}}}{q_n} \right) \tag{28}$$

as inner wall/JCT resistance is changed, the ratio of the distal to outflow resistance changes according to

$$r_d^* = \frac{r_d}{r_d + r_{je} + \widetilde{r_{sc}}} \tag{29}$$

Intraocular blood flow amplitude. We make the assumption that the amplitude of the blood flow into the eye does not depend on intraocular pressure. The amplitude of the ocular blood flow can be estimated according to

$$a_{qb} = \frac{2\pi f a_{ac,N}}{k\,\overline{p_{ac,N}}} \tag{30}$$

Using Equation 6, the value of a_{ac} can then be calculated for a given $\overline{p_{ac}}$ (see §A4.7).

Reference apparent stiffness. Equation 9 relates the apparent stiffness and SC heights to the pressure drop across the inner wall/JCT. This pressure drop can be approximated by

$$\Delta p = \left(\overline{p_{ac}} - \overline{p_{ev}}\right)\left(1 - r_d^*\right) \tag{31}$$

Figure A3 shows h against Δp for the data in Table A3, assuming $r_d^* = r_{d,N}^*$ and $\overline{p_{ev}} = 0$ (as these studies were performed in enucleated eyes). The relationship between h and Δp is clearly nonlinear, and can be characterised by a fit of the form

$$h\left(\Delta p\right) = h_0 e^{-\frac{\Delta p}{\xi_0}} \tag{32}$$

where $\xi_0 = 8.2\ [6.4, 10.0]\ mmHg$ (mean [95% CI]) is the value of ξ at h_0. The reference apparent stiffness, ξ_0, is then used during the iteration procedure to calculate a value of ξ for each value of $\overline{p_{ac}}$ (see §A4.7).

A4.5 *V.* Independent Parameter

The present study uses the *in vivo* paradigm, in which the net flow rate through the system is constant. Therefore, IOP elevation occurs due to an increase in resistance to outflow, the majority of which lies in the vicinity of SC inner wall. The independent parameter in our model is therefore the JCT/inner wall resistance r_{je}.

Cell debris and pigment are transported to the TM by aqueous humour outflow, and phagocytosis by TM cells

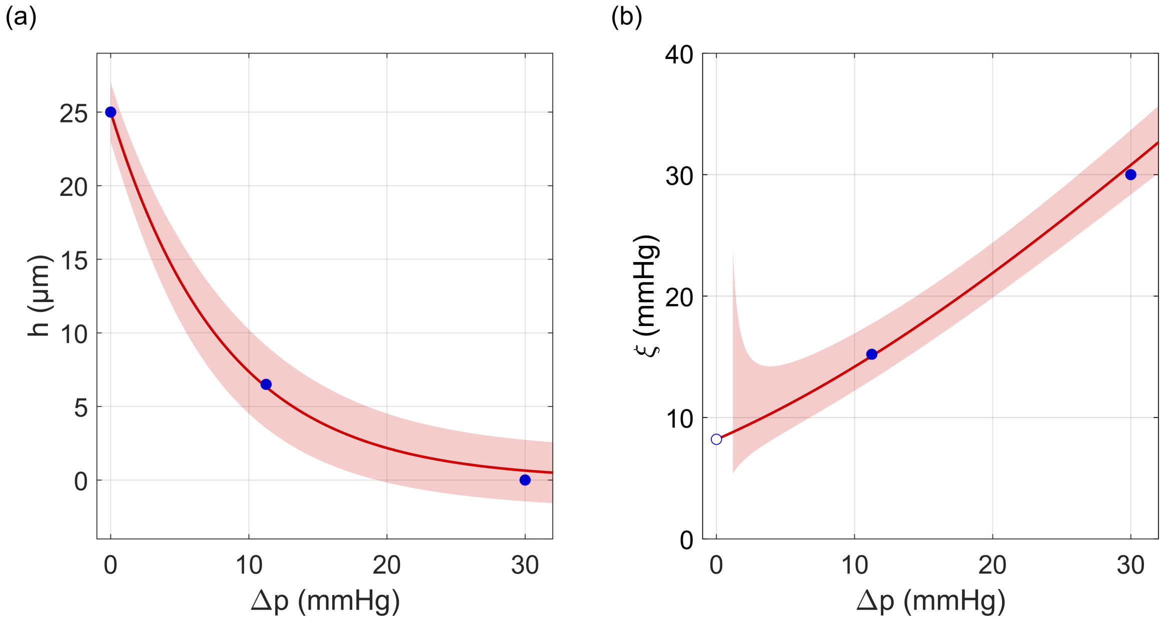

Figure A3: (a) SC height h, as a function of $\Delta p = \overline{p_{ac}} - \overline{p_{sc}}$. Data points show values from Table A3, red curve indicates best fit of Equation 32 to the data and the shaded region indicates the 95% confidence interval. (b) Apparent stiffness as a function of Δp according to Equation 33. The empty marker indicates ξ_0. Filled markers are calculated by applying Equation 9 to values from the second and third rows of Table A3.

is responsible for clearing away this debris. Furthermore, lytic enzymes and extracellular matrix production by TM cells are responsible for matrix remodelling. The extent of debris and matrix accumulation in the TM thereby influences outflow resistance, which we model by varying r_{je}.

For all simulations, r_{je} is varied in the range 0.08 to 7.71 $mmHg/(\mu l/min)$, corresponding to values of p_{ac} between 10.6 and 28.0 $mmHg$ for the 'normal' parameters. A value of $r_{je} = 2.08$ $mmHg/\mu l/min$ corresponds to the 'normal' inner wall/JCT resistance.

A4.6 *VI.* Investigated Parameter

TM stiffness is thought to increase in glaucoma [17, 18]. We model this change by multiplying the apparent stiffness, ξ by a factor n_ξ. We consider $n_\xi = 1.0$, 1.5 and 4.

A4.7 *VII.* Iterated Parameters

For each iteration (see §A3), $\widetilde{r^j_{sc}}$, is updated and used to estimate a new value of $\overline{p_{ac}}$ (Equation 3). A number of the model parameters are dependent on $\overline{p_{ac}}$, and therefore must be updated for each iteration j. Having

calculated $\overline{p_{ac}^j}$ using Equations 3 and 27, Δp^j can be calculated using Equations 29 and 31. The apparent stiffness can be approximated by combining Equations 9 (averaged in space and time) and 32 to yield

$$\xi^j = n_\xi \left(\frac{\Delta p^j}{1 - e^{-\frac{\Delta p^j}{\xi_0}}} \right) \tag{33}$$

where the term n_ξ has been introduced to model increased stiffness, as described in §A4.6. Figure A3b demonstrates the pressure dependence of the apparent stiffness, which increases almost four-fold as Δp increases from 0 to 30 $mmHg$. Subsequently, we can calculate the SC compliance ϕ_{sc}^j (see Table A1), and the ocular pulse amplitude a_{ac}^j (Equation 6). The non-dimensional parameters, can then be calculated according to the relationships given in Table A1.

A5 Numerical Solution

Equation 15 is a non-linear second-order partial differential equation. In order to solve numerically, we define $\theta(X,T) = H^4(X,T)$. Numerical solutions for $\theta(X,T)$ were then found using MATLAB's *pdepe* solver using a relative tolerance of 10^{-4}. The system was solved for 50 cardiac cycles, at which point the root mean square residual of the SC height at the ostium ($X = 1$) was consistently less than 10^{-4}, indicating a converged periodic solution. The final cardiac cycle was then extracted for analysis.

Sensitivity analysis was carried out on mesh resolution, timestep resolution and q_{thresh}. The results showed that the system is sensitive to q_{thresh}, and relatively insensitive to timestep and mesh size. Based on this analysis, a dimensionless mesh size of 0.001 and a dimensionless timestep size of 0.005 were selected with $q_{\text{thresh}} = 2.16$ nl/min (which is 0.1% of $q_{n,N}$). The relaxation factor λ was initially set to 0.7, and in order to avoid oscillations was increased to 0.95 when the sign of $q_n - q_n^j$ changed relative to $q_n - q_n^{j-1}$.

A6 Evaluation of Flat Plate Approximation for Shear Stress

The magnitude of shear stress calculated using Equation 23 is based on the assumption that the flow in SC can be modelled as flow between two flat plates, yielding a dimensional reference shear stress, $\tau_{sc,0} = 6\mu q_0/wh_0^2$. This requires the assumption that $w \gg h$, which is appropriate given that $w = 12h_0$ and the width-to-height ratio increases further as intraocular pressure increases and SC collapses. Had SC been considered as a rectangular channel, the shear stress relative to the flat plate solution would be given by [19]:

$$\frac{\tau(y)}{\tau_{sc,0}} = \frac{8w}{\pi^2\,(w - 0.63h_0)} \sum_{n,odd}^{\infty} \frac{1}{i^2} \left[1 - \frac{\cosh\left(\frac{i\pi y}{h_0}\right)}{\cosh\left(\frac{i\pi w}{2h_0}\right)}\right] \tag{34}$$

where $-w/2 \leq y \leq w/2$. At $y = 0$, the summation reduces to $\pi^2/8$ [19]. Hence, $\frac{\tau(0)}{\tau_{sc,0}} = \frac{w}{w-0.63h_0} = 1.055$, i.e. the peak shear stress is 5.5% higher than that for the flat plate model. This increase results from the larger centreline velocity necessary to make up for the lower velocities near the channel edges, so that the net flow rates are the same for both cases. Although the shear stress drops to zero at the channel edges, and the average shear stress integrated over the inner wall is within 1% of the flat plate model solution.

References

[1] Johnson MC, Kamm RD. The role of Schlemm's canal in aqueous outflow from the human eye. Invest Opthalmol Vis Sci. 1983;24(3):320–325.

[2] Reitsamer HA, Kiel AW. A rabbit model to study orbital venous pressure, intraocular pressure, and ocular hemodynamics simultaneously. Invest Opthalmol Vis Sci. 2002;43(12):3728–3734.

[3] Zamora DO, Kiel JW. Topical proparacaine and episcleral venous pressure in the rabbit. Invest Opthalmol Vis Sci. 2009;50(6):2949–2952.

[4] Rohen JW, Lütjen E, Bárány E. The relation between the ciliary muscle and the trabecular meshwork and its importance for the effect of miotics on aqueous outflow resistance: A study in two contrasting monkey species, Macaca irus and Cercopithecus aethiops. Albrecht von Graefes Arch Klin Exp Ophthalmology. 1967;172(1):23–47.

[5] Rohen JW, Futa R, Lütjen-Drecoll E. The fine structure of the cribriform meshwork in normal and glaucomatous eyes as seen in tangential sections. Invest Opthalmol Vis Sci. 1982;21(4):574–585.

[6] Brubaker RF. Flow of aqueous humor in humans [The Friedenwald Lecture]. Invest Opthalmol Vis Sci. 1991;32(13):3145–3166.

[7] Bill A, Phillips CI. Uveoscleral drainage of aqueous humour in human eyes. Exp Eye Res. 1971;12(3):275–281.

[8] Dietlein TS, Jacobi PC, Lüke C, Krieglstein GK. Morphological variability of the trabecular meshwork in glaucoma patients: implications for non-perforating glaucoma surgery. Brit J Ophthalmology. 2000;84(12):1354–1359.

[9] Moses RA. The effect of intraocular pressure on resistance to outflow. Surv Ophthalmology. 1977;22(2):88–100.

[10] Dvorak-Theobald G. Further studies on the canal of Schlemm: Its anastomoses and anatomic relations. Am J Ophthalmology. 1955;39:165–89.

[11] Kaufmann C, Bachmann LM, Robert YC, Thiel MA. Ocular pulse amplitude in healthy subjects as measured by dynamic contour tonometry. Arch Ophthalmology. 2006;124(8):1104–1108.

[12] Coleman DJ, Trokel S. Direct-recorded intraocular pressure variations in a human subject. Arch Ophthalmology. 1969;82(5):637–640.

[13] McBain EH. Tonometer calibration. II. Ocular rigidity. AMA Arch Ophthalmology. 1958;60(6):1080–91.

[14] Johnson M. What controls aqueous humour outflow resistance? Exp Eye Res. 2006;82(4):545–557.

[15] Allingham RR, de Kater AW, Ethier CR. Schlemm's canal and primary open angle glaucoma: correlation between Schlemm's canal dimensions and outflow facility. Exp Eye Res. 1996;62(1):101–110.

[16] Van Buskirk EM. Anatomic correlates of changing aqueous outflow facility in excised human eyes. Invest Opthalmol Vis Sci. 1982;22(5):625–632.

[17] Last JA, Pan T, Ding Y, Reilly CM, Keller K, Acott TS, et al. Elastic modulus determination of normal and glaucomatous human trabecular meshwork. Invest Opthalmol Vis Sci. 2011;52(5):2147–6.

[18] Wang K, Johnstone MA, Xin C, Song S, Padilla S, Vranka JA, et al. Estimating human trabecular meshwork stiffness by numerical modeling and advanced OCT imaging. Invest Opthalmology Vis Sci. 2017;58(11):4809–4817.

[19] Bruus H. Theoretical Microfluidics. Oxford University Press, UK; 2008.
